# Gender stereotypes in preschool: An examination of age and gender differences

**DOI:** 10.1111/bjdp.70048

**Published:** 2026-05-11

**Authors:** Johanna Landvogt, Jan Lenhart

**Affiliations:** ^1^ University of Bamberg Bamberg Germany

**Keywords:** gender development, gender differences, gender stereotypes, pre‐school children

## Abstract

Gender stereotypes are prevalent in many societies and begin to emerge in early childhood. The present study examined the gender stereotypes and their association with age and gender in preschool. Gender stereotypes of feminine, masculine and gender‐neutral traits and behaviours were collected in a sample of 104 German and Austrian children (52% girls) aged 3–6 years, using a 5‐point rating scale. As expected, older children provided more gender‐stereotypical responses. This association across age resulted primarily from an increase in extreme stereotypical and a decrease in counter‐stereotypical associations. When comparing the gender stereotypes of boys and girls of different ages, younger girls already showed stronger gender stereotypes, while older girls showed more gender stereotype flexibility than boys at the same age. These findings provide new insights into preschool boys' and girls' gender stereotypes.

## INTRODUCTION

Asked to describe which toys and activities boys or girls favour in kindergarten, ChatGPT answers: ‘In kindergarten, boys often enjoy building sets, action figures, and vehicles, while girls typically favor dolls, arts and crafts, and play kitchens’. Even though artificial intelligence like ChatGPT and its use are part of a modern and progressive society, apparently, it can cling to very traditional and not so progressive gender stereotypes (Gross, 2023). Gender stereotypes refer to beliefs about characteristics and traits that men and women possess (descriptive aspect) or should possess (prescriptive aspect) (Eagly, 1987; Fiske & Stevens, 1993). Gender stereotypes affect people's lives in many ways, such as clothing selection (Blakemore, 2003), the pursuit of hobbies (Boiché et al., 2014), interest in school subjects (Li et al., 2022) and career aspirations (Janssen & Backes‐Gellner, 2016).

Apart from this anecdotal proof of gender stereotypes in artificial intelligence, numerous studies show that gender stereotypes are still prevalent in many societies (Eagly et al., 2020; Haines et al., 2016) and that even very young children have already internalized some beliefs about gender (Bian et al., 2017; Halim et al., 2024). For example, even 3‐ to 5‐year‐old children consider appearance to be more important for girls than for boys, which mirrors many adults' beliefs (Halim et al., 2024) and 6‐year‐old girls rate their ability to work on challenging tasks that require them to be ‘really, really smart’ lower than boys (Bian et al., 2017). Although several studies found an increase in gender stereotypes during the preschool years (e.g., Kanka et al., 2014; Miller et al., 2009), our understanding of the mechanisms by which this development occurs and whether there are differences between boys and girls is still incomplete. Accordingly, in the present study, we examined whether gender associations of typically feminine and typically masculine traits and behaviours differ depending on children's age and gender.

### The development of gender stereotypes

Several different theoretical accounts of gender development have been proposed (see Blakemore et al., 2008, for an overview). Besides biological approaches, these accounts can be divided into two schools of thought. So‐called cognitive theories argue that cognitive factors are primarily responsible for the formation of gender stereotypes and gender‐typed behaviour (e.g., Bem, 1981; Kohlberg, 1966; Liben & Bigler, 2002; Martin & Halverson, 1981). So‐called social theories, in contrast, emphasize the influence of social factors (i.e., the social environment) for the development of gender stereotypes and respective behaviour (e.g., Bussey & Bandura, 1999; Eagly & Wood, 2012). Irrespective of their respective focus, these theoretical perspectives highlight that children actively construct gender from both the social environment and their cognitions about the sexes (see Martin & Ruble, 2004).

Concerning developmental patterns of gender stereotypes, Trautner et al. (2005) propose three ordered phases. First, a phase of learning about gender‐related characteristics, mainly taking place during the preschool years. Second, a consolidation of the newly acquired gender knowledge in a rigid either‐or fashion, reaching its peak of rigidity between 5 and 7 years. Third, a phase of relative flexibility following the peak of rigidity. According to Trautner et al. (2005), these phases are theoretically linked to processes of construction (Phase 1), consolidation (Phase 2), and deployment and integration (Phase 3) of gender schemas, which have been proposed as a key element for gender development in gender schema theories (Bem, 1981; Liben & Bigler, 2002; Martin & Halverson, 1981). Gender schemas represent people's ‘organized networks of mental associations representing information about themselves and the sexes’ (Martin et al., 2002, p. 911), which influence information processing and behaviour.

Empirically, several studies have demonstrated that children's knowledge of gender associations increases with age (e.g., Kanka et al., 2014; Leinbach et al., 1997; Miller et al., 2009; see Signorella et al., 1993, for a meta‐analysis of older studies). For instance, Miller et al. (2009) asked 3‐ to 10‐year‐old children to provide open‐ended descriptions of girls and boys. They found that older children provided a higher number of gender stereotypes in their answers. A similar picture emerged when children were asked to choose an adult figure (male or female) for gender‐associated activities such as cooking, fixing things, feeding babies and gender‐associated occupations such as working as a babysitter or a pilot (Albert & Porter, 1983, 1986, 1988). In another study (Leinbach et al., 1997), children aged 4, 5 and 7 years were asked to assign conventional and metaphorical items, mostly objects and some qualities, like angry or soft, to girls or boys or both girls and boys by putting a card with a picture of it in the respective box. Four‐year‐old children gave significant less stereotypic answers than 7‐year‐old children. In a second study, the authors compared the responses of the 4‐year‐olds to those of 3‐year‐olds, with the younger children providing significantly fewer stereotypic responses than their older peers. Finally, in a German‐speaking sample, children aged 3 to 7 years were asked to assign feminine and masculine objects and behaviour patterns to boys, girls or both boys and girls (Kanka et al., 2014). They found that gender‐stereotyped responses increased for both masculine and feminine items with age.

Taken together, there is robust evidence that children's gender stereotypes or, put in other words, their gender associations, which correspond to feminine or masculine associations in society, increase during the preschool years. However, as pointed out by Trautner et al. (2005), who examined the development of gender stereotypes in 5‐ to 10‐year‐old children, the prevalent use of forced‐choice measures (dichotomously either ‘for boys’ or ‘for girls’) or a restriction to three categories (‘for boys’, ‘for girls’ and ‘for both boys and girls’) makes it hard to track developmental changes. For example, if children can select only from ‘for boys’ or ‘for girls’, it is only possible to examine whether children's assignment matches the direction of adults' perception. If the third category (‘for both boys and girls’) is included, children can show gender‐neutral attitudes. However, the differentiation might not be fine enough as traits, objects and activities might be only somewhat associated with masculinity or femininity. In this case, children must choose between two imperfect alternatives, namely to categorize either as gender‐neutral or as only ‘for boys’ or ‘for girls’. To address this issue, following Trautner et al.'s (2005) approach, we used a 5‐point rating scale for measuring stereotypes, rather than a forced‐choice or a 3‐point rating scale, to identify age‐related changes in responding.

### Differences between boys and girls in the development of gender stereotypes

Although this general pattern of an increase in gender stereotypes and of three consecutive phases is expected for both boys and girls (Martin & Ruble, 2004; Trautner et al., 2005), some theoretical accounts propose differences in the development of gender stereotypes between boys and girls. The cognitive development theory (Kohlberg, 1966), for instance, assumes that the development of gender stereotypes is primarily driven by children's general cognitive development. Although the pace of development seems to be similar in boys and girls for many cognitive skills (Ardila et al., 2011), some studies found that girls' language development progresses somewhat faster on average than boys' (Bouchard et al., 2009; Eriksson et al., 2012; Halpern, 2012; Rinaldi et al., 2023). As children's language development is closely related to their cognitive development (Bornstein et al., 2006; Marchman & Fernald, 2008; for a review, see Deák, 2014), it is possible that girls also display gender stereotypes at a younger age than boys. However, from a social perspective, boys typically face more repercussions than girls do if they deviate from gender norms (Freeman, 2007; Kane, 2006; Leaper, 1994). This could lead to boys developing earlier and stronger stereotypes about gender than girls. Finally, according to schema theories (e.g., Martin & Halverson, 1981), children should develop more elaborated schemas for objects and activities that pertain to their own sex because they tend to engage with those objects and pursue those activities. According to this assumption, boys should develop more knowledge about things associated with masculinity, and girls about things associated with femininity.

Empirically, the research picture is decidedly mixed on gender differences. Concerning developmental patterns, Trautner et al. (2005) could not identify distinct developmental patterns for 5‐ to 10‐year‐old boys and girls, with the timing of the peak of rigidity and the level of the peak of rigidity being unrelated to children's gender. However, there is also some evidence for gender differences. Miller et al. (2009), for example, found that girls reported more gender stereotypes than boys from preschool to fifth grade when they were asked to provide open‐ended descriptions of girls and boys. A similar finding was reported by Baker et al. (2016) for gender stereotyping of masculine and feminine occupations. Moreover, Halim et al. (2017) examined 4‐ and 5‐year‐old preschoolers and found that among the 4‐year‐old children, girls answered more often than boys in line with common gender stereotypes and that the development of gender stereotypes was different for boys and girls. In their study, boys' knowledge about gender increased from age 4 to 5, whereas girls' knowledge remained stable in this time span, resulting in similar scores at the age of 5. Finally, a meta‐analytic review of older studies (Signorella et al., 1993) found that girls provide more stereotypic responses in forced‐choice formats (dichotomous answers: male vs. female or boy vs. girl). In contrast, in the case of non‐forced‐choice questions, there were no differences between preschool boys and girls. However, in elementary school, girls even demonstrated a tendency to answer in a less stereotypical manner than boys.

When masculine and feminine items were examined separately, some studies reported stronger stereotypes for feminine items in girls and stronger stereotypes for masculine items in boys (Ebert et al., 2024; Leinbach et al., 1997; Solbes‐Canales et al., 2020), while the overall prevalence of stereotypes did not differ significantly between boys and girls (Leinbach et al., 1997; Solbes‐Canales et al., 2020). Other studies reported corresponding findings only for girls, who indicated stronger stereotypes for feminine items, whereas there was no difference between boys and girls for masculine items (Kanka et al., 2014).

In summary, as already pointed out, the empirical evidence is mixed. Although the available evidence does not support the notion that boys display stronger gender stereotypes, there are some indications that children have stronger gender associations for activities, traits and objects that are associated with their own sex and that girls might acquire gender stereotypes somewhat earlier than boys.

### The current study

In the present study, we built on previous research on the development of gender stereotypes in early childhood (e.g., Martin & Ruble, 2004; Trautner et al., 2005). Although there is robust evidence that children's gender stereotypes increase during the preschool years (e.g., Kanka et al., 2014; Miller et al., 2009) and some studies indicate that it might be worthwhile to examine gender differences as well as feminine and masculine items separately (e.g., Leinbach et al., 1997; Solbes‐Canales et al., 2020), our knowledge about the mechanisms by which these age differences occur in preschool and whether there are differences for boys and girls is still incomplete.

To address this issue, we used a 5‐point scale (‘only boys’, ‘mostly boys’, ‘equally boys and girls’, ‘mostly girls’, ‘only girls’) for assessing children's gender stereotypes for stereotypically feminine, masculine and gender‐neutral items in an age‐diverse group of preschool children. Following Trautner et al.'s (2005) proposition of a learning phase and a subsequent consolidation phase of gender schemata in preschool, we expected older children to answer more strongly in line with the gender stereotypes than younger children (Hypothesis 1). Whereas Trautner et al. (2005) aggregated the answers on the scale in a rigidity score (‘only girls/boys’) and a flexibility score (‘mostly girls/boys’ and ‘equally’), we also looked separately at the use of each of the categories of the 5‐point scale. This is important because an increase in gender stereotypes (i.e., rigidity) could result from a slowly increasing strength of gender stereotypes (i.e., from ‘equally boys and girls’ to ‘mostly girls’ to ‘only girls’), from a jump from gender‐neutral (‘equally boys and girls’) to a strong gender stereotype (‘only girls’) and/or from a jump from gender‐atypical (‘only boys’) to gender‐typical stereotypes (‘only girls’). Similarly, gender flexibility could result from a slowly decreasing strength of stereotypes (i.e., from ‘only girls’ to ‘mostly girls’ to ‘equally boys and girls’) or from a jump from strong gender stereotypes (‘only girls’) to (‘equally boys and girls’).

Another important but still not fully answered question is whether there are differences regarding the age‐related differentiation of gender stereotypes between boys and girls. Based on theoretical considerations about general cognitive developmental trends (Kohlberg, 1966), children focus on cues pertaining to their own sex (Martin & Halverson, 1981), and previous empirical research (e.g., Halim et al., 2017; Leinbach et al., 1997; Solbes‐Canales et al., 2020) we tested the hypotheses that preschool girls show gender stereotypes at an earlier age than their male peers (Hypothesis 2) and that children report stronger gender stereotypes for activities and traits that are associated with their own sex (Hypothesis 3). Finally, to explore children's reasoning about gender stereotypes, we asked a sub‐sample of 10 children for selected items to explain their gender stereotypes.

## METHOD

The study was approved by the Ethics Committee of the University of Bamberg, Germany. Parents received written information about the study in advance and were asked to return a written informed consent for their children's participation. Only children for whom parents had provided written consent were allowed to participate. The data and scripts are available on OSF: https://osf.io/4k87f/overview?view_only=c0f0812799bd4dae895ac4111ce6681c.

### Sample

The initial sample consisted of 106 children. As two children chose not to participate, the final sample consisted of 104 three‐ to six‐year‐old children (54 girls; age between 38 and 83 months, *M* = 61.66, *SD* = 11.14, of whom 11 were three years, 33 four years, 38 five years and 21 six years old; a histogram of the age distribution is included in the electronic supplement). The children were recruited from 10 kindergartens in Germany and Austria. Most parents were born in Germany or Austria, with only 10% (of all cases, 19 missings) of the mothers and 7% (of all cases, 19 missings) of the fathers reporting a different country of birth. The sample's demographic characteristics may be subject to self‐selection bias, as only children whose parents gave consent and were able to read the German information were able to participate. Around half of the sample (51% (of all cases, 20 missings) of the mothers and 47% (of all cases, 24 missings) of the fathers) reported a university entrance qualification or a university degree as their highest level of education.

Based on previous research (Kanka et al., 2014; Leinbach et al., 1997; Miller et al., 2009; Solbes‐Canales et al., 2020; Trautner et al., 2005), we assumed at least a small to medium effect size (*r* ≈ .25 to .30) for correlations with age and for differences between boys and girls. A sensitivity analysis with G*Power (3.1.9.7; Faul et al., 2007, 2009) indicated that our sample size of 104 children was sufficient to detect bivariate correlations greater than *r* = .24, within‐subjects differences (t‐tests for two dependent samples) of greater than *d* = 0.24 and between‐subjects differences (t‐tests for two independent samples) of greater than *d* = 0.49.

### Instruments

#### Parent questionnaire

The parents completed a questionnaire on demographic information, including the child's date of birth, child's gender, child's and parents' land of birth, parents' highest educational levels and languages spoken at home.

#### Children's gender stereotypes

Children's gender stereotypes were assessed with 56 items, comprising 25 feminine, 21 masculine and 10 gender‐neutral words. Each item was answered on a 5‐point scale, consisting of five pictures, ranging from only boys to mostly boys, equally boys and girls, mostly girls and only girls (depicted by 4 boys, 3 boys and 1 girl, 2 boys and 2 girls, 1 boy and 3 girls, and 4 girls). To obtain this set of items, we used a two‐step procedure. First, the childlex database (Schroeder et al., 2015), which contains words from German children's books, was searched for the most common words that represent characteristics, traits or behaviour of persons. This resulted in 894 feminine, masculine and gender‐neutral items that were assumed to be familiar to preschool children. In a second step, a study with adults was conducted to identify words that are clearly associated with femininity and masculinity or that are perceived as gender neutral. Following Bem's concept of psychological androgyny (Bem, 1974), we differentiated between associations with masculinity and femininity. We used separate 5‐point scales asking about the association with femininity and masculinity for each item, ranging from very unfeminine (1) to very feminine (5) and from very unmanly (1) to very masculine (5). To reduce participants' load, the list was split into two lists comprising 447 words and into two versions (masculine or feminine associations), with participants being randomly assigned to the versions. The four questionnaires were administered online via SoSci Survey (Leiner, 2019), whereby 138 subjects (86% female) participated, resulting in 28 to 44 completed questionnaires per condition. Only words that adults rated as very masculine (*M* > 4) and very unfeminine (*M* < 2), as well as those they rated as very feminine (*M* > 4) and very unmanly (*M* < 2), were selected as masculine and feminine items for the present study. In addition, a small selection of 10 words that were neither feminine nor masculine according to both masculinity and femininity ratings (*M* near 3 in both scales) was included as neutral control words (see Table S1).

### Procedure

The study was conducted in the kindergartens, using a separate quiet room, and each child was tested individually. The study was carried out by trained psychology students. One to two sessions were conducted for each child; some children conducted other tests in between, which are not relevant for this study and therefore not reported. Data collection took between 15 and 30 min per session. At the beginning, each child was introduced to the 5‐point scale. After ensuring that the children understood the scale, the first 28 words were presented to the children. Oriented at Trautner et al. (2005) we asked them ‘Can you show me who does/is …’. The children answered the question by pointing to the appropriate picture on the scale. They were instructed as follows: For the end of the scale (only boys or girls), children were told to point to these pictures if only girls or boys do or are something (‘only’). For the next category (one boy or girl and three girls or boys), they were told to point here if it was something that girls or boys mostly do or are, but that boys or girls do or are as well sometimes (‘mostly’). Lastly, for the middle category (two boys and two girls), they were told to point here if it was something that girls and boys do or are equally often (‘equally’). If the children could not answer immediately, the word was repeated, followed by an explanation of the word. If the children were still unable to answer, the word was coded as missing. Then the children were asked if they needed a break. The experimenter could also decide to take a break or to do the second half of the experiment in another session if it became clear that a child was no longer able to concentrate. The second half of the study was conducted in the same way as described above.

Finally, to obtain a better understanding of children's reasons for their gender associations, all participants in the last kindergarten (a sub‐sample of 10 children, 5 boys and 5 girls; of whom 9 completed the task, aged 42 to 72 months, *M* = 57.30, *SD* = 11.70) were asked follow‐up questions (why do you think [behaviour/attitude] is [repeated individual answer of the child in the first part, i.e. only for boys/girls, mostly for boys/girls, equally for both?]), on 8 items. To avoid overtaxing the children, we chose only to ask for explanations for 8 items. The items were selected to investigate the children's answers regarding different aspects. As we observed that there were words to which many of the children did not respond as expected, we included those words to obtain a better understanding of the children's reasons to answer not in line with the adults (to turn, to shriek and innocent). However, we also included two masculine words, two feminine words and a gender‐neutral word for which most of the children answered as expected (beautiful, to wash, to fight, powerful and to sink).

### Data analysis

Data preparation was conducted with IBM SPSS 31.0, and the analysis with JASP 0.95.4.0. The missing data rates for children's gender stereotypes were low (*M* = 4% per word, range 0% ‐ 17% per word), and age was only missing for one child. The missing per child varied between 0 and 26. The number of missing's per child did not vary significantly with age (*r* = −.13, *p* = .184; 95% CI [−0.32, 0.06]) or sex (*r* = −.12, *p* = .221; 95% CI [−0.31, 0.07]). Accordingly, we chose not to impute any data on the item level. Instead, missing values were addressed on the scale level by averaging across the available items of the masculine, feminine and neutral set of items. To obtain a single score for children's gender stereo typicity, the answers on the masculine and feminine categorized items were recoded into a scale ranging from anti‐stereotypic (−2) to stereotypic (2), with 0 representing a neutral rating (feminine items: 5 → 2, 4 → 1, 3 → 0, 2 → −1, 1 → −2; masculine items: 5 → −2, 4 → ‐1, 3 → 0, 2 → 1, 1 → 2). Then the means of the masculine and feminine items were calculated. The general stereotypicity score was calculated by averaging the means of the female and male stereotypicity scores. The significance threshold was set at *p* < .05 and all directional hypotheses were tested one‐tailed, with 90% CIs being reported. All other tests were conducted two‐tailed, with 95% CIs being reported.

For the exploratory qualitative analysis, the interviews were transcribed verbatim, except for pauses and dialect. Repetitions and encouraging words after the children's final answers by the interviewer were not transcribed. The analysis followed the procedure advised by Maring (e.g., Mayring, 2022, Qualitative content analysis). The minimal coding units were defined as clear meaning components in the text. Gender, age and the quantitative answer were also included as context information in the analysis. The first author coded the children's answers following an inductive approach, which was then discussed with the second author and revised. An independent second coder recoded the answers to ensure interrater reliability. Interrater reliability was high (*κ* = 0.83, *z* = 14.10, *p* < .001).

## RESULTS

### Preliminary analyses

Before addressing the hypotheses, we checked whether children's gender associations of the words corresponded with the categorization (masculine, neutral, feminine) based on the adult ratings in the pilot study. To do so, we calculated the Wilcoxon signed‐rank tests for each word and tested whether the word significantly differed from a neutral rating (*M* = 3). Most words were categorized according to the adult ratings (95% of the masculine words, 88% of the feminine words and 70% of the gender‐neutral words). Regarding the masculine words, one word was not statistically significantly categorized as predicted (‘broad’), but descriptively, its mean pointed in the expected direction (*M* = 2.80). Three feminine words (‘to sob’, ‘innocent’ and ‘to shriek’) were not statistically significantly rated as feminine by the children. While the mean value of sobbing (*M* = 3.22) pointed in the expected direction, shrieking and innocent were even rated in the masculine direction (*M* = 2.52 and *M* = 2.76). Three of the 10 words, which were expected to be gender neutral, deviated significantly from a neutral rating (‘silly’, ‘to turn around’ and ‘to turn’), with silly (*M* = 2.65) being rated in the masculine direction and the other two words in the feminine direction (*M* = 3.37 and *M* = 3.50). In the electronic supplement, there is an overview of adults' ratings (Table S1) as well as a detailed description of children's ratings for each word (Table S2). In the subsequent analyses, we used only the words that significantly corresponded to the adults' rating. The remaining 20 masculine items were statistically significantly rated as masculine (*M* = 2.32, *SD* = 0.75; *t*(103) = −9.29, *p* = <.001, *d* = 0.74, 95% CI [−1.14; −0.68]) and the remaining 22 feminine items as feminine (*M* = 3.67, *SD* = 0.58; *t*(103) = 11.81, *p* = <.001, *d* = 0.58, 95% CI [0.91; 1.41]).

As can be seen in Table 1 the children used the categories of the 5‐point rating scale with different frequencies. The ‘stereotypic’ answer was the most often used category on the 5‐point scale (46%), whereas both ‘mostly’ categories were rarely used (11% and 8%). A separate inspection of the feminine and masculine items indicated that the distribution of the items was similar for masculine and feminine items. In addition, the use of the different categories was similar for girls and boys.

**TABLE 1:** Children's answers on the 5‐point scale.

Item	Stereotypic	Mostly stereotypic	Equally	Mostly counter‐stereotypic	Counter‐stereotypic
Total sample					
All items (%)	1938 (46%)	457 (11%)	914 (22%)	331 (8%)	576 (14%)
Masculine (%)	918 (45%)	216 (11%)	391 (23%)	174 (7%)	273 (14%)
Feminine (%)	1020 (47%)	241 (11%)	523 (20%)	157 (9%)	305 (14%)
Girls					
All items (%)	1004 (46%)	244 (11%)	473 (22%)	185 (8%)	269 (12%)
Masculine (%)	444 (48%)	127 (10%)	202 (23%)	98 (7%)	142 (11%)
Feminine (%)	560 (44%)	117 (13%)	271 (20%)	84 (10%)	127 (14%)
Boys					
All items (%)	934 (46%)	213 (10%)	441 (22%)	149 (7%)	309 (15%)
Masculine (%)	474 (42%)	89 (11%)	189 (23%)	76 (7%)	131 (16%)
Feminine (%)	460 (50%)	124 (9%)	252 (20%)	73 (8%)	178 (14%)

*Note*: All items stand for all feminine and masculine items which were rated in the expected direction (42 in total).

### Gender stereotypes across age

The first hypothesis proposed a significant increase in gender stereotypes with age. As expected, we found a positive correlation between children's age and the degree of stereotypicity (*r* = .27, *p* = .003, 90% CI [.11, .41]). This pattern replicated when the stereotypicity score was examined separately for feminine items (*r* = .23, *p* = .010, 90% CI [.07, .38]) and masculine items (*r* = .24, *p* = .008, 90% CI [.08, .38]). These findings support the first hypothesis, showing an age‐related increase in gender stereotypes during the preschool years.

In addition, to test for non‐linear associations with age (i.e., an onset of gender flexibility), a quadratic term for age was added to the linear predictor age (mean‐centred). There was no significant incremental variance explained, when the quadratic term for age was added to the linear models (all items: Δ*R* ^2^ = .02, *B* = −0.00, *SE* = 0.00, *β* = −0.12, *t* = −1.29, *p* = .100, 90% CI for *B* [−0.00, 0.00]; feminine items: Δ*R* ^2^ = .01, *B* = −0.00, *SE* = 0.00, *β* = −0.09, *t* = −0.95, *p* = .172, 90% CI for *B* [−0.00, 0.00]; masculine items: Δ*R* ^2^ = .01, *B* = −0.00, *SE* = 0.00, *β* = −0.12, *t* = −1.24, *p* = .109, 90% CI for *B* [−0.00, 0.00]), providing no indication of a beginning gender flexibility in the overall sample.

Then we inspected the correlations between age and each of the categories of the 5‐point scale. There was a significant increase for ‘stereotypic’ answers (*r* = .23, *p* = .009, 90% CI [.07, .38]) and a significant decrease in ‘counter‐stereotypic’ answers (*r* = −.18; *p* = .037, 90% CI [−.33, −.02]) and in ‘mostly counter‐stereotypic’ answers (*r* = −.24; *p* = .015, 95% CI [−.41, −.05]). In contrast, the categories ‘mostly stereotypic’ (*r* = −.09, *p* = .363, 95% CI [−.28, .11]) and ‘equally’ (*r* = .06, *p* = .577, 95% CI [−.14, .25]) did not significantly correlate with age (see Figure S4).

### Gender differences across age

The second hypothesis proposed that girls learn gender stereotypes at an earlier age than boys. To test this hypothesis, a regression analysis was conducted with the stereotypicity score as the criterion variable and age (mean centred), gender and their interaction as predictor variables. The effects of age (mean centred) (*B* = 0.01, *SE* = 0.01, *β* = 0.10, *t* = 0.81, *p* = .210, 90% CI for *B* [−0.01, 0.02]) and gender (*B* = −0.10, *SE* = 0.11, *t* = −0.90, *p* = .186, 90% CI for *B* [−0.28, 0.08]) were not significant. However, the interaction of age and gender was significant (Δ*R* ^2^ = .04, *B* = 0.02, *SE* = 0.01, *t* = 2.07, *p* = .021, 90% CI for *B* [0.00, 0.04]). Follow‐up analyses indicated that there was a significant correlation between age and gender stereotypicity for boys (*r* = .44, *p* < .001, 90% CI [.23, .62]) but not for girls (*r* = .12, *p* = .207, 90% CI [−.12, .33]) (see Figure 1). This pattern replicated when separate analyses were conducted for masculine items (boys: *r* = .37, *p* = .004, 90% CI [.15, .56]; girls: *r* = .11, *p* = .226, 90% CI [−.13, .33]) and feminine items (boys: *r* = .45, *p* < .001, 90% CI [.24, .62]; girls: *r* = .08, *p* = .275, 90% CI [−.15, .31]). Accordingly, as can be seen in Figure 1, the existence of gender differences in gender stereotypicity depended on the age of the children. To analyse the interaction between age and gender further, we used Hayes' Process Macro for SPSS and analysed the conditional effects at the 16th, 50th and 84th percentiles (default setting) with gender as moderator. Girls showed stronger gender stereotypes at 50 months (16% percentile: *B* = −0.35, *SE* = 0.16; *t* = −2.11, *p* = .038, 95% CI for *B* [−0.67; −0.21]) but not at 62 months (50% percentile: *B* = −0.09, *SE* = 0.11; *t* = −0.83, *p* = .407, 95% CI for *B* [−0.31; 0.13]) and 74 months (84% percentile: *B* = 0.16, *SE* = 0.16; *t* = 0.96, *p* = .342, 95% CI for *B* [−0.17; 0.48]). Thus, the findings supported the second hypothesis that girls display higher levels of gender stereotypes already at an earlier age.

**FIGURE 1:**
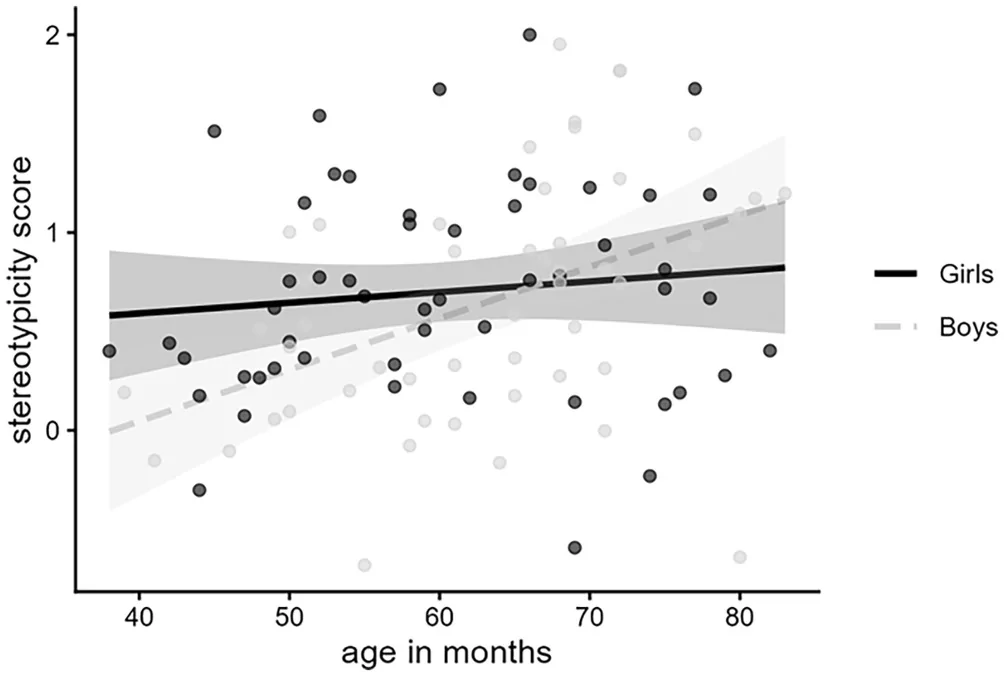
Stereotypicity score across age for boys and girls. 90% confidence intervals are displayed.

To examine if girls and boys also differed regarding the onset of gender flexibility (third phase of the developmental model), we examined separately for girls and boys whether adding a quadratic term for age explained incremental variance in children's gender stereotypicity. While there was a significant linear effect of age (mean centred) on gender stereotypicity in boys (*B* = 0.03, *SE* = 0.01, *β* = 0.44, *t* = 3.40, *p* = <.001, 90% CI for *B* [0.01, 0.04]), adding the quadratic term did not account for additional variance (Δ*R* ^2^ = .00, *B* = −0.00, *SE* = 0.00, *β* = −0.04, *t* = −0.30, *p* = .382, 90% CI for *B* [−0.00, 0.00]). In contrast, there was no significant linear effect of age (mean centred) on the gender stereotypicity score for girls (*B* = 0.00, *SE* = 0.01, *β* = 0.07, *t* = 0.52, *p* = .302, 90% CI for *B* [−0.01, 0.01]), but adding the quadratic term incrementally explained variance (Δ*R* ^2^ = .07, *B* = −0.00, *SE* = 0.00, *β* = −0.27, *t* = −1.93, *p* = .030, 90% CI for *B* [−0.00, −0.00]). Based on the regression equation, the estimated maximum of stereotypicity for girls was at 63 months (see Figure 2).

**FIGURE 2:**
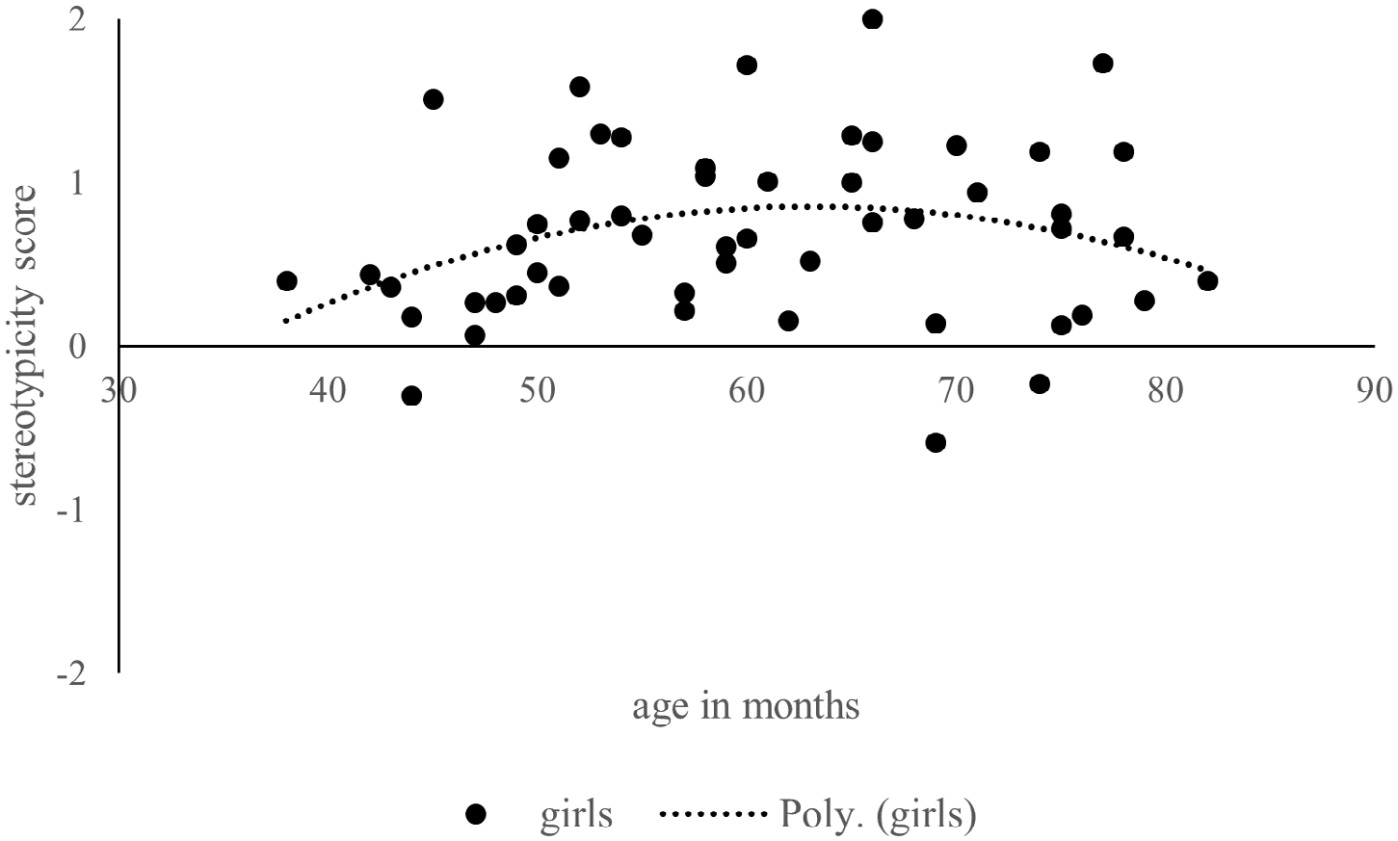
Stereotypicity score for girls across age with a quadratic trendline.

The use of the individual categories on the scale was also analysed separately for boys and girls. Whereas girls showed no significant increase of ‘stereotypic’ answers (*r* = .11, *p* = .226, 90% CI [−.13, .33]) and no significant decrease of ‘counter‐stereotypic’ answers with age (*r* = −.15, *p* = .141, 90% CI [−.37, .08]), boys showed a significant increase in ‘stereotypic’ answers (*r* = .38, *p* = .003, 90% CI [.16, .57]) and a significant decrease in ‘counter‐stereotypic’ answers (*r* = −.24, *p* = .044, 90% CI [−.45, −.01]). In contrast, a significant increase for the ‘equally’ category was found with age for girls (*r* = .27, *p* = .050, 95% CI [.00, .50]) but not for boys (*r* = −.19, *p* = .196, 95% CI [−.44, .10]). For the ‘mostly stereotypic’ answers neither girls (*r* = −.22, *p* = .118, 95% CI [−.46, .06]) nor boys (*r* = .06, *p* = .686, 95% CI [−.22, .33]) showed a significant correlation with age. While the ‘mostly counter‐stereotypic’ category in girls was marginally significant (*r* = −.26 *p* = .057, 95% CI [−.50, .01]), it was not significant for boys (*r* = −.20, *p* = .160, 95% CI [−.45, .08]).

For girls, we additionally analysed whether adding the quadratic term explained additional variance for the categories of the scale. For the ‘stereotypic’ and ‘mostly stereotypic’ answers, adding the quadratic term explained significantly incremental variance (‘stereotypic’: Δ*R* ^2^ = .19, *B* = −0.03, *SE* = 0.01, *β* = −0.43, *t* = −3.32, *p* = .002, 95% CI for *B* [−0.05, −0.01], estimated maximum at 62 months; ‘mostly stereotypic’: Δ*R* ^2^ = .19, *B* = 0.01, *SE* = 0.01, *β* = 0.38, *t* = 2.98, *p* = .004, 95% CI for *B* [0.01, 0.02], estimated minimum at 59 months). For the three other categories, adding the quadratic term did not significantly explain additional variance (‘equally’: Δ*R* ^2^ = .08, *B* = 0.01, *SE* = 0.01, *β* = 0.10, *t* = 0.71, *p* = .484, 95% CI for *B* [−0.01, 0.02]; ‘mostly counter‐stereotypic’: Δ*R* ^2^ = .11, *B* = 0.01, *SE* = 0.00, *β* = 0.21, *t* = 1.59, *p* = .118, 95% CI for *B* [−0.00, 0.01]; ‘counter‐stereotypic’: Δ*R* ^2^ = .03, *B* = −0.00, *SE* = 0.01, *β* = −0.09, *t* = −0.66, *p* = .513, 95% CI for *B* [−0.01, 0.00]).

### Gender differences in masculine and feminine items

To examine the third hypothesis that proposed stronger stereotypes for items associated with children's own sex, we compared boys' and girls' ratings of masculine and feminine items (see Figure 3). There was a significant difference in stereotypicity of masculine and feminine items for boys, *t*(49) = −2.11, *p* = .020, *d* = −0.30, 90% CI [−0.54, −0.06], with boys having stronger stereotypes for masculine words (*M* = 0.74, *SD* = 0.75) than for feminine words (*M* = 0.57, *SD* = 0.61). Although there was no significant difference between masculine and feminine items in girls, *t*(53) = 1.37, *p* = .088, *d* = 0.19, 90% CI [−0.04, 0.41], the stereotypicity was descriptively higher for feminine (*M* = 0.77, *SD* = 0.53) than for masculine items (*M* = 0.63, *SD* = 0.75). In addition, girls reported significantly stronger stereotypes for feminine words than boys, *t*(102) = 1.79, *p* = .038, *d* = 0.35, 90% CI [0.01, 0.39]. In contrast, for masculine words, there was only a descriptive trend favouring stronger stereotypes in boys, which was not statistically significant, *t*(102) = −0.72, *p* = .236, *d* = −0.14, 90% CI [−0.35, 0.14]. Therefore, these findings only partially support the third hypothesis.

**FIGURE 3:**
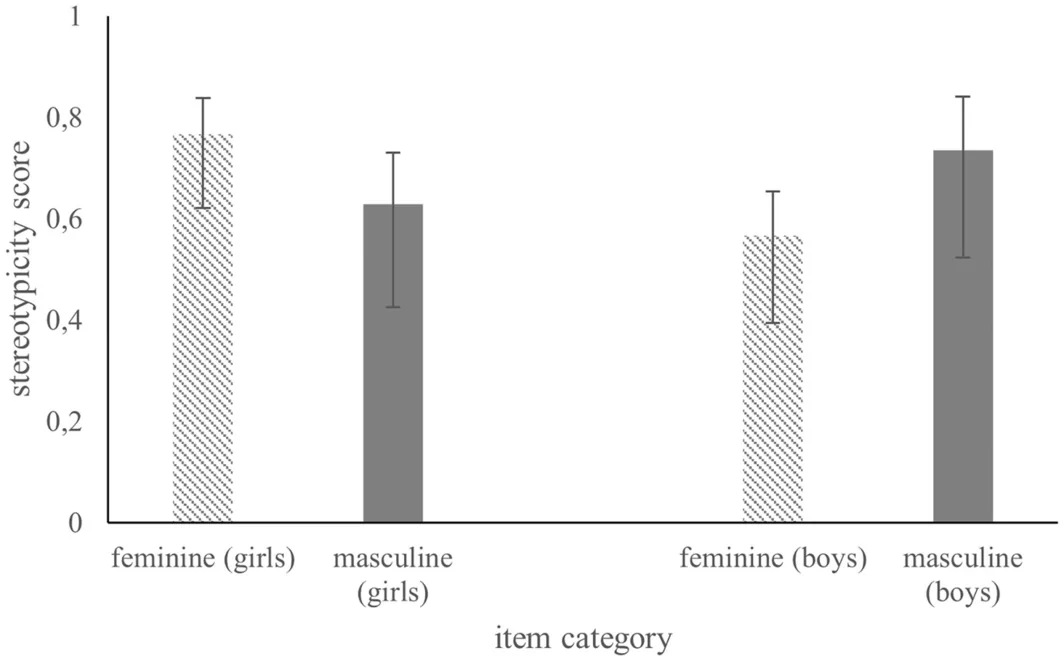
Stereotypicity scores for masculine and feminine items for boys and girls. Error bars represent ± 1 standard error.

### Exploratory qualitative analysis of children's explanations for their gender stereotypes

Following the main study, a small sample of the children was asked about the reasons for their gender‐association ratings. Table 2 provides an overview of the inductively derived categories and examples for each category. In 40% of the time, the children answered ‘I don't know’, gave no answer at all or an answer that was not interpretable or related to the question. In contrast, the ten children showed only two missing quantitative answers (i.e., ratings) on these items, indicating a divergence between the ability to provide a gender association and to explain it. Interestingly, some children provided explanations for almost every item, whereas some children mostly said, ‘I don't know’. Individual differences (shyness/openness, cognitive availability of stereotypes, ability to give explanations, situational factors like tiredness, etc.) have probably influenced whether an answer was provided.

**TABLE 2:** Categories and frequency of explanations in the qualitative analysis.

Research question: What informs children's answers regarding gender stereotypes?			
	Category	Examples	Frequency
A. In line with adult stereotype ratings	*A1*: Generalizing answer; association generally related to characteristics/behaviour of the social group	‘to fight: because boys are so strong.’; ‘beautiful: because boys don't like dresses.’	17%
	*A2*: Referencing to individual behaviour/experience from family or close social environment	‘to sink: because when my mother is cleaning, and she makes an up and down motion.’	7%
	*A3*: No preferential treatment for either sex	‘to turn: while dancing’	3%
B. Different to adult stereotype ratings	*B1*: Generalizing answer; association generally related to characteristics/behaviour of the social group	‘powerful: because girls are strong.’; ‘to turn: because they (girls) wear dresses.’	17%
	*B2*: Referencing to individual behaviour/experience from family or close social environment	‘to wash: both men and women are washing, because I (boy), my mom, my brother and sometimes my father are washing.’	11%
	*B3*: No preferential treatment for either sex	‘beautiful: I can't decide.’; ‘to fight: nobody does that because it hurts.’	6%
C. No answer	*C*: no interpretable statement	‘I forgot.’; ‘I don't know.’	40%

When children provided explanations, those were mostly short, with only a couple of words or a sentence. Around 34% of the explanations comprised generalizations based on behaviour or characteristics of boys/men and girls/women. Another 18% of the explanations referred to children's personal experiences from their own behaviour or their close social environment. Finally, 6% of the explanations were based on no association with persons' sexes. As some words were generally rated incongruent to the adult rating, it was expected that in the subsample, some children would provide counter‐stereotypic ratings and explanations. Interestingly, when looking at children's verbal answers, children provided explanations for words which they answered in line with adult stereotypes nearly as often as for the words they answered not in line with adult stereotypes. The kind of reasoning did not seem to differ for counter‐stereotypical and stereotypical answers. Notably, there was one word which seemed to have a similar meaning for nearly every child. The word ‘to turn’, which is considered a neutral item in the adult rating, was explained very often with answers such as ‘because girls are dancing’ or ‘because girls are wearing a dress’, which implies that for many children to turn indicated dancing, which might have led to the feminine association of the item in the quantitative ratings.

## DISCUSSION

### Summary and interpretation of the results

In this study, we examined age‐related and gender‐associated differences in gender stereotypes in preschool children. Following, Trautner et al.'s (2005) proposition of a three‐phase development, we examined whether older preschool children answer more rigidly in line with gender stereotypes than their younger peers (Hypothesis 1). Importantly, to explore the age‐related differences in greater depth, we used a 5‐point scale, which allows a more fine‐grained examination. We also examined whether girls and boys show different developmental patterns (Hypothesis 2) and whether children have stronger gender stereotypes for activities and traits matching their own sex (Hypothesis 3).

As proposed in Hypothesis 1, older children answered more often in line with adults' gender stereotypes than younger children, indicating an increase in gender stereotypes with age. While this result has to be interpreted with caution as the effect was small to moderate (*r* = .27, one‐tailed), it is in line with previous research on gender stereotype development in children (Kanka et al., 2014; Leinbach et al., 1997; Miller et al., 2009) and supports Trautner et al.'s (2005) proposition that gender stereotypes become increasingly rigid during the preschool years. Interestingly, an analysis of the 5‐point scale indicated that this age‐related difference was primarily driven by an increase in extreme stereotypic answers (‘only for boys’ or ‘only for girls’) and a decrease in extreme counter‐stereotypic answers. In contrast, the use of ‘equally’ as well as the ‘mostly stereotypic/counter‐stereotypic’ answers did not systematically vary with age, and their use was low across all age groups. This indicates that the increasing gender rigidity during the preschool years might result from a direct switch from counter‐stereotypic answers, which occur quite often in young children, to stereotypic answers and not from a gradually increasing association with gender. However, as our data are only cross‐sectional in nature, this is just a reasonable assumption based on the distribution of the categories across age. In future studies, a longitudinal design examining transitions between categories is necessary to test this assumption.

For the second hypothesis, which assumed differences of gender stereotypes for girls and boys, associations between age and gender stereotypicity were analysed separately for boys and girls. In line with the hypothesis, there was an interaction of age and gender. The additional variance explained by the interaction indicated a significant, but small effect. Boys started at a somewhat lower level of gender stereotypes, which increased with age, while girls' gender stereotypes did not significantly increase but remained quite stable across the age span examined in the present study (3–6 years). Accordingly, only younger girls reported significantly more gender stereotypes than their male peers, whereas there were no differences in older preschool children. This pattern is similar to Halim et al.'s (2017) findings and supports the hypothesis that girls' acquisition of gender stereotypes starts somewhat earlier than boys' learning of gender stereotypes. This aligns with findings for language development showing an accelerated development for girls in preschool (Bouchard et al., 2009; Halpern, 2012; Rinaldi et al., 2023) and supports the idea that cognitive development might be an important factor in explaining the development of gender stereotypes (as proposed by Kohlberg, 1966). However, it has to be noted that these differences between boys and girls in gender stereotypes and language skills, such as grammar or vocabulary, might also stem from differences in environmental input. For instance, mothers tend to talk more with their daughters than with their sons (for a meta‐analysis, see Leaper et al., 1998), and adults seem to react more contingently to communicative acts of girls (e.g., Fagot et al., 1985), which might—at least in part—explain differences in grammatical proficiency and vocabulary knowledge but also in children's learning of gender stereotypes. Irrespective of the relevance of cognitive or social factors to this developmental difference, the differences between boys and girls across age might also help to explain why differences in overall gender stereotypes are found in some studies (e.g., Baker et al., 2016), while others report no such overall differences (e.g., Leinbach et al., 1997; Solbes‐Canales et al., 2020).

In addition to the gender difference regarding the onset of gender stereotypes, older girls answered more often ‘equally’ than younger girls, which was not the case for boys. This indicates a beginning flexibility of gender stereotypes at the end of preschool, corresponding to the third stage of the model proposed by Trautner et al. (2005). In line with this, we also found that including a quadratic term for age explained additional variance for girls but not for boys. The peak of rigidity for girls' gender stereotypes (found at 63 months in the present sample) was primarily reflected in the use of the stereotypic category, which first increased and then decreased in girls (peak found at 62 months in the present sample). In addition, at around this age (59 months in the present sample), girls' use of the ‘mostly stereotypic’ category began to increase. Girls' increasing use of the ‘mostly stereotypic’ category indicates a more nuanced perception of gender associations regarding specific behaviours and traits, and therefore more gender flexibility (Trautner et al., 2005). It seems that the rigid ‘only boys/girls’ categories are gradually being replaced by the ‘equally’ and the ‘mostly stereotypical’ categories. In other words, traits and behaviours are increasingly perceived as being equally suitable for both boys and girls or being somewhat associated with femininity/masculinity (as indicated by the ‘mostly stereotypic’ category). These findings strengthen the assumption that cognitive development might differentially drive the timing of the development of gender stereotypes for boys and girls. However, it has to be noted that the differences between girls and boys were small and if older children had been included in our study, we would expect to find a similar pattern of beginning gender flexibility (but at a somewhat later age) for boys (see Trautner et al., 2005).

We also examined whether girls have stronger stereotypes about feminine words and boys about masculine words (Hypothesis 3). Indeed, boys had significantly stronger stereotypes for masculine words than for feminine words. For girls, the descriptive difference also pointed in the expected direction, with feminine items showing descriptively higher stereotypicity ratings. In addition, girls showed stronger gender stereotypes about feminine words than boys. For masculine words, there were no significant gender differences, although boys reported descriptively higher gender stereotypes for masculine words than girls. The effect sizes were small to medium in size. Accordingly, this lends at least partial support to Hypothesis 3. Interestingly, some of the previous studies showed similar results. Kanka et al. (2014) reported stronger stereotypes for feminine items for girls and no difference between boys and girls for masculine items. In the study of Solbes‐Canales et al. (2020), girls showed stronger stereotypes for typically feminine occupations, but there was no difference for typically masculine occupations. This finding might be explained by the fact that girls showed an earlier onset of gender stereotypes, and that only at the end of preschool did boys reach girls' levels of gender stereotypes. The greater difference between masculine and feminine items in boys than in girls and the greater difference between boys and girls in feminine items than in masculine items might therefore stem from gender differences in developmental patterns. Supporting this, we found that the increase in stereotypicity was strongest for feminine items in boys. Finally, the proposition of the schema theories (e.g., Martin & Halverson, 1981) that children should develop more elaborated schemas for objects and activities that pertain to their own sex might primarily apply to the differentiation of gender schema. Boys develop more and deeper knowledge about things associated with masculinity, and girls about things associated with femininity (Martin & Halverson, 1981). They might, however, only to a lesser degree differ regarding the superficial association of certain activities and traits with gender.

Finally, it must be noted that we found that most items already displayed gender associations that corresponded to adults' gender stereotypes. This was to be expected because we made sure that only items were chosen that should be known to the children and which showed either a clear neutral, feminine or masculine association in the adult rating. However, despite the sophisticated selection procedure, some items deviated from this pattern (e.g., shrieking, innocent, to turn). The results of our selective qualitative analysis suggest that children's understanding of some words (e.g., to turn) differs from adults' deeper and more differentiated semantic lexicon. In addition, the qualitative analysis of the subsample's reasons indicated that preschool children are often not able to explain their answers, which mirrors findings from previous studies (Szkrybalo & Ruble, 1999; Wehren & De Lisi, 1983). There seems to be a gap between the quantitative answers (i.e., gender‐association ratings), where the children answered most of the time and the ability to explain the reasoning behind these answers. Wehren and De Lisi (1983) reported, for example, that older children seem to be able to explain their answers more often than younger children, which shows that the ability to explain gender stereotypes may develop with age. When the children in our study provided answers, they used mostly explanations that referred to experiences at home or with other children or that generalized from other traits and behaviours that are associated with boys and girls (e.g., ‘because they are strong’ for explaining their answer for ‘to fight’ or ‘because they are so nice’ for ‘beautiful’). Interestingly, this kind of reasoning was used for both gender‐congruent and gender‐incongruent answers. These findings indicate that—irrespective of their congruence with adults' or society's attitudes—children use both their personal experiences as well as generalizations based on gender categories to explain their gender associations.

### Limitations and directions for future research

Although the present study offers many insights into preschool children's gender stereotypes, some limitations need to be considered. First, as the present study used a cross‐sectional design, it offers indications of possible development paths, but no conclusions can be drawn about intra‐individual development. To do so, a longitudinal design that accompanies children across the preschool years (and in the best case, through primary school) would be necessary. This would also help to gain more clarification regarding the development of the use of the answer categories on the 5‐point scale. Second, our sample of items was limited to a selection of behaviours and traits. It would therefore be interesting to replicate the study with different sets of items. Nevertheless, in our opinion, it is a strength of the present study that we conducted a systematic selection process to obtain age‐appropriate items that are clearly associated with femininity and masculinity. Unfortunately, the present set of items did not allow for a systematic analysis of different areas of gender stereotypes (e.g., characteristics/behaviours regarding appearance, brilliance, etc.). Another limitation is that our sample is not representative of the German or Austrian population because children with a migration background were underrepresented. This kind of self‐selection is a well‐known problem in research with young children. Therefore, we cannot exclude the possibility that our research findings might not generalize to those populations. Further, the reasons for the response behaviour were only explored qualitatively in a small sub‐sample and for a small, selected set of items. To gain insight into children's reasoning about gender appropriateness, a more systematic qualitative approach would be necessary. This could also help to increase item validity, as the qualitative analysis suggests that children might sometimes understand or use words differently from adults.

## CONCLUSION

The results of our study extend our knowledge from previous studies (e.g., Trautner et al., 2005), and deepen the understanding of gender stereotypes across preschool. We could show that the strength of gender stereotypes increases across preschool. Even if the possibility to answer fine‐grained and gradually on a 5‐point scale was provided, preschool children only rarely used the ‘mostly’ categories, showing a clear preference for extreme gender associations. We could also show that it is important to differentiate between boys and girls, as girls showed higher gender stereotypes at an earlier age and started to use the flexible ‘equally’ category earlier than boys. Finally, our qualitative results highlight that children use their close environment, mostly experiences with other children and at home, as well as generalizations of other behaviours and traits associated with gender, to explain their gender associations.

## AUTHOR CONTRIBUTIONS

**Johanna Landvogt:** Conceptualization; investigation; writing – original draft; methodology; visualization; writing – review and editing; formal analysis; data curation. **Jan Lenhart:** Conceptualization; writing – review and editing; methodology; writing – original draft; supervision; resources.

## CONFLICT OF INTEREST STATEMENT

The authors declare that they have no conflicts of interests.

## Supporting information


Table S1.
Table S2.
Figure S3.
Figure S4.

## Data Availability

The data necessary to reproduce the analyses and the study material presented here are publicly accessible (Link: https://osf.io/4k87f/overview?view_only=79d3712eb8084e27b72c93e0519d26fc). The analyses presented here were not preregistered.
