# Supplementary material for: Gender stereotypes in preschool: An examination of age and gender differences
Source: Br J Dev Psychol. 2026 May 11;44(4):771–87. doi: 10.1111/bjdp.70048 (PMC13648301; doi:10.1111/bjdp.70048)
Supplement: Supplementary file 1 [file BJDP-44-771-s001.docx]

**Supplementary Material**

**Table S1**

*Adults‘ ratings of selected items (pilot-study)*

| **Item** | **Item category** | **Feminine rating** | |  | | | **Masculine rating** | |
| --- | --- | --- | --- | --- | --- | --- | --- | --- |
|  |  | *M* | *SD* | |  | *M* | | *SD* |
| fressen (to wolf down food) | m | 1.65 | 0.79 | |  | 4.23 | | 0.77 |
| glitzern (to sparkle) | w | 4.53 | 0.65 | |  | 1.40 | | 0.54 |
| spucken (to spit) | m | 1.54 | 0.82 | |  | 4.12 | | 0.71 |
| angreifen (to attack) | m | 1.66 | 0.64 | |  | 4.40 | | 0.54 |
| umbringen (to murder) | m | 1.74 | 0.74 | |  | 4.02 | | 0.60 |
| schluchzen (to sob) | w | 4.29 | 0.63 | |  | 1.48 | | 0.67 |
| mischen (to mix) | n | 3.03 | 0.47 | |  | 3.00 | | 0.38 |
| vorsichtig (catious) | w | 4.12 | 0.59 | |  | 1.95 | | 0.58 |
| drohend (threatening) | m | 1.78 | 0.83 | |  | 4.33 | | 0.53 |
| süß (sweet) | w | 4.58 | 0.72 | |  | 1.68 | | 0.69 |
| backen (to bake) | w | 4.45 | 0.68 | |  | 1.65 | | 0.74 |
| schlagen (to beat) | m | 1.52 | 0.63 | |  | 4.43 | | 0.55 |
| rosa (pink) | w | 4.77 | 0.43 | |  | 1.23 | | 0.42 |
| ängstlich (anxious) | w | 4.19 | 0.54 | |  | 1.67 | | 0.53 |
| verschwinden (to vanish) | n | 3.00 | 0.85 | |  | 3.03 | | 0.86 |
| tanzen (to dance) | w | 4.52 | 0.63 | |  | 1.75 | | 0.67 |
| bauen (to build) | m | 1.83 | 0.80 | |  | 4.53 | | 0.64 |
| sanft (gentle) | w | 4.66 | 0.55 | |  | 1.68 | | 0.66 |
| unschuldig (innocent) | w | 4.28 | 0.75 | |  | 1.98 | | 0.62 |
| donnern (to thunder) | m | 1.93 | 0.84 | |  | 4.08 | | 0.66 |
| **Item** | **Item category** | **Feminine rating** | |  | | | **Masculine rating** | |
|  |  | *M* | *SD* | |  | *M* | | *SD* |
| schwach (weak) | w | 4.10 | 0.77 | |  | 1.56 | | 0.79 |
| albern (silly) | n | 3.04 | 0.85 | |  | 3.00 | | 0.85 |
| kräftig (strong) | m | 1.69 | 0.76 | |  | 4.53 | | 0.68 |
| umdrehen (to turn around) | n | 3.00 | 0.38 | |  | 3.03 | | 0.58 |
| kreischen (to shriek, to squeal) | w | 4.45 | 0.69 | |  | 1.48 | | 0.78 |
| umarmen (to embrace) | w | 4.31 | 0.76 | |  | 1.93 | | 0.62 |
| kochen (to cook) | w | 4.38 | 0.73 | |  | 1.83 | | 0.64 |
| hilflos (helpless) | w | 4.03 | 0.68 | |  | 1.83 | | 0.75 |
| heulen (to wail) | w | 4.52 | 0.63 | |  | 1.35 | | 0.62 |
| überlassen (to leave to) | n | 2.95 | 0.67 | |  | 3.00 | | 0.86 |
| marschieren (to march) | m | 1.45 | 0.63 | |  | 4.53 | | 0.60 |
| putzen (to clean) | w | 4.38 | 0.73 | |  | 1.65 | | 0.74 |
| breit (broad) | m | 1.97 | 0.98 | |  | 4.18 | | 0.64 |
| glänzen (to shine) | w | 4.03 | 0.82 | |  | 1.98 | | 0.70 |
| drohen (to threaten) | m | 1.76 | 0.69 | |  | 4.14 | | 0.76 |
| kämpfen (to fight) | m | 1.93 | 0.96 | |  | 4.62 | | 0.57 |
| klemmen (being stuck) | n | 2.96 | 0.56 | |  | 3.00 | | 0.89 |
| brüllen (to yell, to shout) | m | 1.89 | 0.80 | |  | 4.12 | | 0.65 |
| weinen (to cry) | w | 4.44 | 0.71 | |  | 1.41 | | 0.67 |
| brav (well-behaved) | w | 4.26 | 0.75 | |  | 1.90 | | 0.62 |
| bedrohlich (menacing) | m | 1.70 | 0.70 | |  | 4.19 | | 0.51 |
| waschen (to wash) | w | 4.04 | 0.56 | |  | 1.85 | | 0.67 |
| **Item** | **Item category** | **Feminine rating** | |  | | | **Masculine rating** | |
|  |  | *M* | *SD* | |  | *M* | | *SD* |
| wunderschön (beautiful) | w | 4.32 | 0.84 | |  | 1.85 | | 0.67 |
| schwenken (to swifel, to wave) | n | 3.00 | 0.55 | |  | 3.00 | | 0.65 |
| weich (soft) | w | 4.05 | 0.79 | |  | 1.75 | | 0.55 |
| bärtig (bearded) | m | 1.29 | 0.56 | |  | 4.90 | | 0.31 |
| jagen (to hunt) | m | 1.71 | 0.72 | |  | 4.65 | | 0.49 |
| befinden (are located/ found) | n | 3.09 | 0.42 | |  | 3.00 | | 0.00 |
| mächtig (powerful) | m | 1.81 | 0.68 | |  | 4.65 | | 0.49 |
| gewaltig (tremendous) | m | 1.95 | 0.80 | |  | 4.15 | | 0.59 |
| kümmern (to take care) | w | 4.10 | 0.77 | |  | 1.90 | | 0.72 |
| senken (to lower) | n | 3.00 | 0.63 | |  | 3.05 | | 0.69 |
| töten (to kill) | m | 1.90 | 1.04 | |  | 4.05 | | 0.69 |
| fühlen (to feel) | w | 4.24 | 0.77 | |  | 1.80 | | 0.52 |
| zerstören (to destroy) | m | 1.90 | 0.54 | |  | 4.35 | | 0.59 |
| drehen (to turn) | n | 2.95 | 0.50 | |  | 3.00 | | 0.46 |

**Table S2**

*Children’s rating of the selected items*

|  | **Adults’ rating** | ***M*** | ***SD*** | **Med** | ***Z*** | ***p*** | ***N*** | ***r*** |
| --- | --- | --- | --- | --- | --- | --- | --- | --- |
| **jagen (to hunt)** | m | 2.00 | 1.28 | 1 | -6.05 | <.001 | 99 | -.61 |
| **bärtig (bearded)** | m | 2.01 | 1.43 | 1 | -6.05 | <.001 | 102 | -.60 |
| **kämpfen (to fight)** | m | 2.01 | 1.40 | 1 | -5.84 | <.001 | 103 | -.58 |
| **kräftig (strong)** | m | 2.02 | 1.34 | 1 | -5.60 | <.001 | 101 | -.56 |
| **töten (to kill)** | m | 2.03 | 1.42 | 1 | -5.45 | <.001 | 86 | -.59 |
| **schlagen (to beat)** | m | 2.14 | 1.25 | 2 | -5.56 | <.001 | 101 | -.55 |
| **gewaltig (tremendous)** | m | 2.16 | 1.47 | 1 | -5.15 | <.001 | 97 | -.52 |
| **bauen (to built)** | m | 2.23 | 1.37 | 2 | -4.93 | <.001 | 99 | -.50 |
| **angreifen (to attack)** | m | 2.26 | 1.50 | 2 | -4.44 | <.001 | 102 | -.44 |
| **donnern (to thunder)** | m | 2.38 | 1.52 | 2 | -3.73 | <.001 | 95 | -.38 |
| **drohend (threatening)** | m | 2.38 | 1.50 | 2 | -3.81 | <.001 | 104 | -.37 |
| **drohen (to threaten)** | m | 2.40 | 1.41 | 2 | -4.08 | <.001 | 99 | -.41 |
| **zerstören (to destroy)** | m | 2.40 | 1.51 | 2 | -3.63 | <.001 | 100 | -.36 |
| **brüllen (to yell, to shout)** | m | 2.47 | 1.47 | 2 | -3.62 | <.001 | 104 | -.35 |
| **spucken (to spit)** | m | 2.48 | 1.56 | 2 | -3.01 | .001 | 98 | -.30 |
| **mächtig (powerful)** | m | 2.50 | 1.61 | 2 | -3.08 | .001 | 100 | -.31 |
| kreischen (to shriek, to squeal) | f | 2.52 | 1.55 | 2 | -2.90 | .002 | 102 | -.29 |
| **umbringen (to murder)** | m | 2.58 | 1.64 | 2 | -2.60 | .005 | 93 | -.27 |
| **marschieren (to march)** | m | 2.62 | 1.39 | 3 | -2.59 | .005 | 100 | -.26 |
| albern (silly) | n | 2.65 | 1.62 | 3 | -2.31 | .021^a^ | 102 | -.23 |
| **bedrohlich (menacing)** | m | 2.69 | 1.54 | 3 | -2.11 | .018 | 96 | -.22 |
| **fressen (to wolf down food)** | m | 2.75 | 1.52 | 3 | -1.80 | .036 | 93 | -.19 |
| unschuldig (innocent) | f | 2.76 | 1.56 | 3 | -1.44 | .075 | 99 | -.14 |
|  | **Adults’ rating** | ***M*** | ***SD*** | **Med** | ***Z*** | ***p*** | ***N*** | ***r*** |
| **verschwinden (to vanish)** | n | 2.79 | 1.54 | 3 | -1.58 | .114^a^ | 100 | -.16 |
| breit (broad) | m | 2.80 | 1.52 | 3 | -1.57 | .058 | 101 | -.16 |
| **schwenken (to swifel, to wave)** | n | 2.90 | 1.50 | 3 | -0.62 | .534 ^a^ | 97 | -.06 |
| **klemmen (being stuck)** | n | 2.95 | 1.53 | 3 | -0.47 | .642 ^a^ | 99 | -.05. |
| **mischen (to mix)** | n | 2.98 | 1.36 | 3 | -0.38 | .706^a^ | 103 | -.04. |
|  | | | | | | | | |
| **senken (to lower)** | n | 3.02 | 1.49 | 3 | -0.09 | .925 ^a^ | 96 | -.01 |
| **befinden (are located/ found)** | n | 3.04 | 1.46 | 3 | 0.22 | .827 ^a^ | 95 | 0.02 |
| schluchzen (to sob) | f | 3.22 | 1.50 | 3 | 1.44 | .076 | 102 | .14 |
| **überlassen (to leave to)** | n | 3.23 | 1.41 | 3 | 1.66 | .097 ^a^ | 98 | .17 |
| **kochen (to cook)** | f | 3.32 | 1.56 | 3 | 2.08 | .019 | 103 | .20 |
| **heulen (to wail)** | f | 3.34 | 1.45 | 3 | 2.13 | .017 | 103 | .20 |
| umdrehen (to turn around) | n | 3.37 | 1.48 | 3 | 2.35 | .019 ^a^ | 100 | .24 |
| **backen (to bake)** | f | 3.42 | 1.49 | 3 | 2.62 | .004 | 102 | .26 |
| **hilflos (helpless)** | f | 3.45 | 1.54 | 4 | 2.89 | .002 | 101 | .29 |
| **kümmern (to take care)** | f | 3.45 | 1.52 | 3 | 2.79 | .003 | 100 | .28 |
| **fühlen (to feel)** | f | 3.46 | 1.38 | 3 | 3.13 | .001 | 98 | .32 |
| **ängstlich (anxious)** | f | 3.47 | 1.45 | 3 | 3.16 | .001 | 104 | .31 |
| drehen (to turn) | n | 3.50 | 1.47 | 3 | 3.35 | <.001^a^ | 100 | .34 |
| **schwach (weak)** | f | 3.50 | 1.60 | 4 | 3.16 | .001 | 101 | .31 |
| **weich (soft)** | f | 3.52 | 1.51 | 4 | 3.34 | <.001 | 100 | .33 |
| **weinen (to cry)** | f | 3.53 | 1.38 | 3 | 3.64 | <.001 | 102 | .36 |
|  | **Adults’ rating** | ***M*** | ***SD*** | **Med** | ***Z*** | ***p*** | ***N*** | ***r*** |
| **süß (sweet)** | f | 3.59 | 1.54 | 4 | 3.63 | <.001 | 101 | .36 |
| **sanft (gentle)** | f | 3.64 | 1.47 | 4 | 3.67 | <.001 | 102 | .36 |
| **vorsichtig (catious)** | f | 3.67 | 1.39 | 4 | 4.33 | <.001 | 103 | .43 |
| **brav (well-behaved)** | f | 3.69 | 1.45 | 4 | 4.20 | <.001 | 103 | .41 |
| **umarmen (to embrace)** | f | 3.69 | 1.35 | 4 | 4.76 | <.001 | 102 | .47 |
| **waschen (to wash)** | f | 3.80 | 1.32 | 4 | 5.52 | <.001 | 102 | .55 |
| **glänzen (to shine)** | f | 3.90 | 1.38 | 5 | 5.44 | <.001 | 102 | .54 |
| **wunderschön (beautiful)** | f | 3.99 | 1.34 | 5 | 5.86 | <.001 | 102 | .58 |
| **tanzen (to dance)** | f | 4.14 | 1.31 | 5 | 6.71 | <.001 | 103 | .66 |
| **glitzern (to sparkle)** | f | 4.21 | 1.20 | 5 | 7.27 | <.001 | 104 | .71 |
| **rosa (pink)** | f | 4.41 | 1.11 | 5 | 8.08 | <.001 | 104 | .79 |

*Note:* If not noted differently, one-tailed significance is reported; a: two-tailed significance is reported; words in bold were rated by the children as expected

**Figure S3**

*Distribution of participants across age*

| **S4a** |   **S4b** |
| --- | --- |
| **S4c** | **S4d** |
|   **S4e** |  |

**Figure S4**

*Frequencies of answer categories across age*

*Note.* 95% CIs are displayed; S4a: Frequency of stereotypic answers; S4b: Frequency of mostly stereotypic answers; S4c: Frequency of equally stereotypic answers; S4d: Frequency of mostly counter-stereotypic answers; S4e: Frequency of mostly counter-stereotypic answers
